# Preoperative optimization of modifiable risk factors is associated with decreased superficial surgical site infections after total joint arthroplasty: a prospective case-control study

**DOI:** 10.2340/17453674.2024.41012

**Published:** 2024-07-17

**Authors:** Maria SIGURDARDOTTIR, Martin Ingi SIGURDSSON, Rafael Daniel VIAS, Yngvi OLAFSSON, Ingibjorg GUNNARSDOTTIR, Emil L SIGURDSSON, Sigurbergur KARASON

**Affiliations:** 1 Department of Anaesthesia and Intensive Care, Landspitali – The National University Hospital of Iceland, Reykjavik; 2 Faculty of Medicine, University of Iceland, Reykjavik; 3 Department of Mathematics, Faculty of Physical Sciences, University of Iceland, Reykjavik; 4 Department of Orthopeadics, Landspitali – The National University Hospital of Iceland, Reykjavik; 5 Department of Clinical Nutrition, Landspitali – The National University Hospital of Iceland, Reykjavik; 6 Faculty of Food Science and Nutrition, University of Iceland; 7 Development Centre for Primary Health Care, Reykjavik, Iceland

## Abstract

**Background and purpose:**

The aim of our study was to investigate change in modifiable risk factors following preoperative optimization and whether risk of superficial surgical site infection (SSI) after total joint arthroplasty (TJA) could be reduced.

**Methods:**

This is a prospective study of implementation of a preoperative optimization pathway for patients waiting for primary TJA. Information regarding the intervention arm was collected from January 2019 to January 2021, first at decision for operation and then at preoperative assessment 1 week prior to operation. The control arm was included between August 2018 and September 2020 after receiving conventional preoperative preparation and information gathered at preoperative assessment. Follow up occurred 6 weeks postoperatively for both groups. The primary outcome was postoperative superficial SSI.

**Results:**

The optimization effort resulted in improvement of weight, anemia, HbA1c, vitamin D, and patient engagement. At preoperative assessment the baseline characteristics of the 2 groups were similar except that the intervention group had substantially more comorbidities. Regarding superficial SSI, association was found with BMI ≥ 30 and HbA1c ≥ 42 mmol/mol in the control group but not in the intervention group. When corrected for differences in ASA classification (reflecting comorbidities), age, and sex, being in the intervention group was associated with lower odds of occurrence of superficial SSI compared with the control group (OR 0.64, 95% confidence interval 0.42–0.97).

**Conclusion:**

We showed that preoperative optimization in a structured cooperation between hospital and primary care was associated with a reduced risk of superficial SSI.

There are several well-known modifiable risk factors that increase risk of periprosthetic joint infections (PJI) after total joint arthroplasty (TJA). These include anemia, dysglycemia/diabetes, obesity, poor nutritional status, smoking [1] and possibly reduced physical capacity [2].

There is increasing interest in preoperative optimization to decrease risk of postoperative complications, especially infections as they reduce quality of life substantially for patients and increase costs extensively [3,4].

The structure of such preoperative optimization programs has varied and they are either hospital or primary care centered or include cooperation between the 2 organizations [5-7]. The choice of which risk factors are optimized varies considerably, as well as what target is set to be obtained [8]. Little has been published regarding the design, implementation, or efficacy of such programs [9,10].

The aim of our study was to evaluate change in modifiable risk factors after preoperative optimization. Moreover, we investigated its association with the incidence of postoperative superficial surgical site infection (SSI) compared with conventional preparation.

## Methods

This is a prospective case control study evaluating outcome of an intervention effort to improve patients’ modifiable risk factors as compared with conventional preoperative preparation before primary TJA at a single institution. The study was conducted according to the STROBE guidelines for observational studies.

The routines of TJA surgery, nature of information gathered, variables collected, definitions used, and how patients were categorized have previously been described [11]. In short, data collection included age, gender, weight, comorbidities, surgery and anesthesia type, ASA classification, various laboratory variables, blood transfusions, and length of stay. BMI was classified according to the WHO’s definition, encompassing different weight categories. Diabetes status was determined by preoperative diagnosis or HbA1c levels. Non-diabetic patients were considered to have dysglycemia if HbA1c was 42– 47 mmol/mol and undiagnosed diabetes if HbA1c > 47 mmol/mol. Preoperative anemia was defined based on WHO’s criteria for hemoglobin concentration (< 120 g/L in women and < 130 g/L in men). Patients at risk of malnutrition were identified by low serum albumin or lymphocyte count. Vitamin-D deficiency was defined by 25-OH-D concentration < 50 nmol/L. Due to costs, only patients with vitamin-D value < 50 nmol on decision for operation had a repeated measurement 1 week prior to operation. Engagement, physical activity, smoking history, and program satisfaction was assessed with questionnaires.

### Intervention group, optimization of patient pathway

We started in 2017 to improve modifiable risk factors utilizing the waiting time of patients before TJA at Landspitali, the National University Hospital of Iceland. Support of hospital and primary health care directors was gained, and agreement made with primary care physicians. Information meetings were held at the various primary health care centers involved. An optimization patient pathway was designed and routes of communication between parties decided, including primary care physicians, hospital orthopedic outpatient clinic, preoperative assessment center, department of clinical nutrition, and specialist outpatient services.

After a pilot trial, inclusion in the intervention started in January 2019 and continued until January 2021, and the patients were operated on from March 2019 to December 2022. The COVID-19 pandemic caused some disruption of planning.

The patients were offered participation 6–12 months prior to surgery at the hospital orthopedic outpatient clinic after being referred there by their primary care physician. If willing to participate, baseline blood tests were taken, and BMI registered. An appointment with their primary care physician was booked within 3 weeks to evaluate results of blood test and assess whether further tests, treatments, or consultations were necessary, focusing on obesity, glycemic control, anemia, nutritional status, smoking cessation, and exercise.

Patients with BMI ≥ 40 received a phone call from a hospital-based clinical nutritionist and were offered a personal interview where general advice regarding food intake was given. Patients with vitamin D < 50 nmol/L received 1 phone call from a hospital-based nutritionist with recommendations for daily doses of vitamin-D supplements based on serum concentration value and weight with the aim to reach a concentration of 75 nmol/L prior to operation, preferably within 3 months [12]. Patients were instructed to continue to take the supplements until the operation. Risk of malnutrition was assessed using a validated screening tool [13] and those defined to be at risk received a consultation.

1 week prior to surgery the patients were re-evaluated at the hospital preoperative assessment clinic, and were asked to answer a questionnaire regarding anemia treatment, nutrition, smoking, physical activity, involvement of healthcare providers in the months preceding the operation, will to influence modifiable risk factors, and awareness of the preoperative optimization efforts and satisfaction with the process.

### Control group

The patients in the control group were already on the waiting list for TJA when the study started and had received conventional preoperative preparation, which consisted of general recommendations from the orthopedic surgeon regarding weight, smoking, and lifestyle on decision for operation, with no further follow-up. Inclusion occurred between August 2018 and September 2020 at the hospital outpatient preoperative assessment clinic 1 week prior to surgery. If they accepted participation in the study the same evaluation occurred as in the intervention group and a questionnaire was answered.

### Follow-up

Follow up was the same for both groups. Patients who had a total hip arthroplasty (THA) were assessed 2–3 weeks postoperatively in primary care for suture removal and evaluation of surgical site complications, while all total knee arthroplasty (TKA) patients were referred to the hospital orthopedic outpatient clinic as they were considered at higher risk of SSI. All patients were offered a 6-week postoperative follow-up visit at the hospital orthopedic outpatient clinic. Patient records from primary care and hospitals were reviewed to identify diagnoses of superficial or deeper surgical site infections and other complications. Superficial SSIs were diagnosed according to CDC criteria based on clinical features of peri-incisional pain, tenderness, swelling, erythema, heat, and antibiotic prescriptions [14]. Other recorded complications were drainage, bleeding, dehiscence, and hematoma. Deeper SSIs or periprosthetic joint infections (PJI) involving fascia, muscle layers, and prostheses were diagnosed by orthopedic surgeons using validated criteria and confirmed with 5 tissue cultures during a DAIR operation (debridement, antibiotics, and implant retention) [15].

### Statistics

Group sizes were evaluated after analyzing prevalence of superficial SSI in the first 100 patients in the control group identifying 8 patients diagnosed with superficial SSI. These results were presented at the congress of the Icelandic Society of Anesthesia and Intensive Care Medicine, 2019. We estimated that optimization of modifiable risk factors could reduce superficial SSIs from 8% to 4%, and this would require patient groups of 550 in each arm for comparisons, but our initial plan was to reach 1,000.

A Shapiro–Wilk test was used to assess normality of data. The Newcombe Score method was used to compute confidence intervals for the differences in proportions in 2-sample and paired-sample nominal data. When comparing differences in mean values of 2-sample and paired-sample continuous data, a Gaussian assumption was in no case reasonable, thus, bias-corrected and accelerated bootstrap confidence intervals were computed. When evaluating the differences between the groups the reference value for the significance of the 95% confidence interval (CI) is zero.

A univariate logistic regression analysis was used to measure association between patient characteristics and the occurrence of superficial SSI in the intervention group. A multivariable logistic regression analysis was used to measure association between the intervention and superficial SSI while adjusting for age, sex, and ASA classification. When evaluating the odds ratios the reference value for the significance of the 95% CI is 1.

The data analysis was done using R (version 4.2.2; R Foundation for Statistical Computing, Vienna, Austria). Missing data was below 3% in all clinical variables. In answers to questionnaires, missing data were between 2.4% and 65.9%. No imputation was performed.

### Ethics, registration, funding, and disclosures

The study was approved by the Science Committee of the Capital Area’s Primary Care and the University of Iceland and by the Icelandic National Bioethics Committee (case number: VSN-18-098) and registered at ClinicalTrials.gov (NCT05399186). Support was provided by grants from the Landspitali Research Fund (A-2019-056, A-202-042, A-2021-036) and the Research Fund of Sigridur Larusdottir by the University of Iceland. Data sharing is possible upon reasonable request. The authors declare no conflict of interest. Complete disclosure of interest forms according to ICMJE are available on the article page, doi: 10.2340/17453674.2024.41012

## Results

Between August 2018 and December 2022, 2,611 patients received a primary TJA in our department. Between August 2018 and September 2020, 744 patients accepted to participate in in the control group but 6 were excluded, thus 738 patients or 69% of eligible TJA patients were included. Between January 2019 and January 2021, 1,010 patients accepted to participate in the intervention arm but 264 were excluded, mostly (n = 259) because they declined operation when this was to be scheduled. Thus, 746 patients were included, or 43% of eligible TJA patients (Figure).

**Figure:**
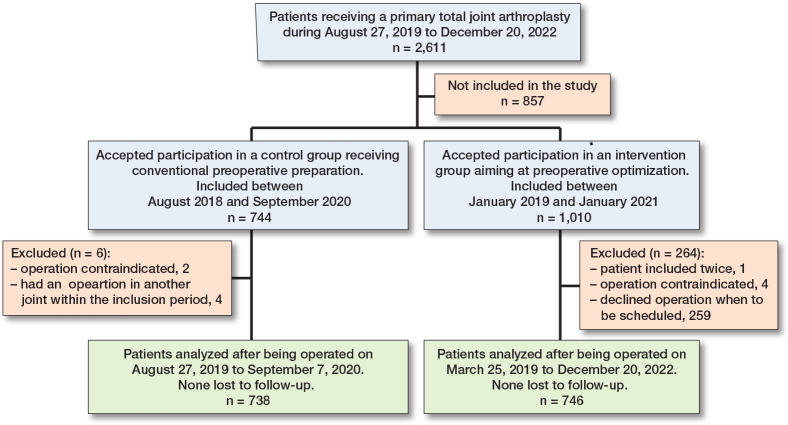
Patient flow chart

### Optimization in the intervention group

The mean length of wait for operation for the intervention group was 356 days (standard deviation [SD] 223). Mean BMI decreased slightly (difference –0.4, CI –0.6 to –0.3) but most notably in those with BMI ≥ 40 on decision for operation (difference –2.4, CI –3.9 to –1.0). Among patients with BMI ≥ 40, 90% received consultation regarding weight loss (40 from a hospital-based clinical nutritionist, 11 from primary care, 6 underwent bariatric surgery, 4 were treated at centers with weight loss programs). Other indices that improved were anemia (difference –1.5, CI –3.4 to 0.4), HbA1c (difference –0.4, CI –0.7 to –0.1), and vitamin D (difference 34.7, CI 28.6 to 41.5), where 92% of those with deficiency received consultation and increased their mean value from 36 to 71 nmol/L 1 week before operation (difference 34.7 CI 28.6 to 41.5) (Table 1).

**Table 1:** Intervention group, patient characteristics (N = 746) at 2 timepoints: on decision for operation and 1 week prior to it. Difference between the 2 timepoints is calculated between means when presented, otherwise between proportions (shown in parentheses) and is presented with 95% confidence interval. Values are count (%) unless otherwise specified

Patient characteristics of intervention group	On decision for operation	Missing	1 week prior to operation	Missing	Difference [CI]
Days waiting ^a^			355 (223)	0 (0)	
Age ^a^			67.9 (9.7)	0 (0)	
Female sex			414 (55)	0 (0)	
BMI ^a^	31.4 (5.8)		31.0 (5.4)		–0.4 [–0.6 to –0.3]
< 18.5	1 (0.1)		1 (0.1)		0.0 [–0.5 to 0.5]
18.5–24.9	94 (13)		102 (14)		1.1 [–0.3 to 2.5]
25–29.9	228 (31)		231 (31)		0.4 [–1.8 to 2.6]
30–34.9	236 (32)		241 (32)		0.7 [–1.8 to 3.1]
35–39.9	124 (17)		126 (17)		0.3 [–2.0 to 2.5]
40–44.9	46 (6.2)		39 (5.2)		–0.9 [–2.5 to 0.6]
45–49.9	15 (2.0)		6 (0.8)		–1.2 [–2.4 to –0.3]
≥ 50	2 (0.3)		0 (0)		
Nutritionist consultation ^b^					
BMI ≥ 40	63 (8.4)		45 (6.0)		–2.4 [–3.9 to –1.0]
Hemoglobin, g/L ^a^	141.4 (12.8)	1 (0.1)	140.9 (12.6)		–0.6 [–1.2 to 0.1]
Anemia	55 (7.4)	1 (0.1)	44 (5.9)	0 (0)	–1.5 [–3.4 to 0.4]
Mild	46 (6.2)		38 (5.1)		–1.1 [–3.1 to 0.9]
Moderate	9 (1.2)		6 (0.8)		–0.4 [–1.5 to 0.6]
Severe	0 (0)		0 (0)		0.0 [–0.5 to 0.5]
Creatinine, µmol/L ^a^	82.7 (41.6)	2 (0.3)	83.9 (47.7)		1.1 [0.1 to 2.2]
Glucose, mmol/L ^a^	6.0 (1.3)	2 (0.3)	6.4 (1.5)	1 (0.1)	0.3 [0.2 to 0.4]
HbA1c, mmol/mol ^a^	38.2 (7.6)	3 (0.4)	37.8 (7.3)	7 (0.9)	–0.4 [–0.7 to –0.1]
HbA1c for non-DM patients		2 (0.3)		7 (1.1)	
42–47 mmol/mol	46 (6.2)		49 (6.6)		0.5 [–1.7 to 2.7]
> 47 mmol/mol	6 (0.8)		8 (1.1)		0.3 [–0.7 to 1.4]
Albumin, g/L ^a^	45.0 (2.7)	3 (0.4)	43.4 (2.7)	10 (1.3)	–1.6 [–1.8 to –1.4]
Albumin < 35 g/L	1 (0.1)	3 (0.4)	1 (0.1)	10 (1.3)	0.0 [–0.6 to 0.6]
Lymphocytes < 1.5x10⁹/L	106 (14)	1 (0.1)	79 (11)	1 (0)	–3.6 [–6.3 to–1.0]
Vitamin D, nmol/L ^a^	84.7 (34.5)	5 (0.7)			
Nutritionist consultation ^b^					
Vitamin D < 50 on					
decision, nmol/L ^a^	36.2 (9.3)		70.9 (28)		34.7 [28.6 to 41.5]

a Values are mean (SD)

b Individuals who had BMI ≥ 40 and those with vitamin D < 50 nmol/L on decision for operation received a nutritionist consultation on decision for operation. The change in number of patients with BMI ≥ 40 is shown at the 2 different timepoints and the change in mean vitamin D for the group that had < 50 nmol/L on decision for operation.

### Comparison of intervention and control group

The 2 groups were similar regarding age, sex ratios, BMI distribution, type of operation and anesthesia, length of stay, and laboratory values. However, the intervention group had a higher ratio of several preoperative comorbidities in general, such as various heart diseases, anticoagulation treatment, renal insufficiency, and the ratio of diabetes was also higher in the intervention group, or 12.5% versus 8.8% (difference 3.7%, CI 0.5–6.8). This was reflected in a higher ratio of patients with ASA ≥ 3 classification or 32% versus 23% (difference 9.1%, CI 4.6–13.6). Postoperative medical complications within the first 6 weeks after operation were generally uncommon in both groups and no deaths occurred (Table 2).

**Table 2:** Comparisons of parameters of control and intervention groups 1 week prior to operation, perioperatively and 6 weeks postoperatively. Difference between the 2 groups is calculated between means when presented, otherwise between proportions (shown in parentheses) and is presented with 95% confidence interval. Values are count (%) unless otherwise specified

Patient characteristics 1 week prior to operation	Intervention group (n = 746)	Missing	Control group (n = 738)	Missing	Difference [CI]
Age ^a^	67.9 (9.7)		66.7 (8.8)		1.2 [0.3 to 2.2]
Female sex	414 (55)		421 (57)		–1.6 [–6.6 to 3.5]
BMI ^a^	31.0 (5.4)		30.6 (5.2)	1 (0.1)	0.4 [–0.2 to 0.9]
< 18.5	1 (0.1)		1 (0.1)		0.0[–0.6 to 0.6]
18.5–24.9	102 (14)		94 (13)		0.9 [–2.5 to 4.4]
25–29.9	231 (31)		258 (35)		–4.0 [–8.8 to 0.7]
30–34.9	241 (32)		238 (32)		0.0 [–4.7 to 4.8]
35–39.9	126 (17)		108 (15)		2.2 [–1.5 to 5.9]
40–44.9	39 (5.2)		33 (4.5)		0.8 [–1.5 to 3.0]
45–49.9	6 (0.8)		5 (0.7)		0.1 [–0.9 to 1.1]
≥ 50	0 (0)		0 (0)		0.0 [–0.5 to 0.5]
Patient comorbidities					
Smoking	28 (3.8)	10 (1.3)	54 (7.3)	11 (1.5)	–3.6 [–6.1 to –1.3]
Hypertension	465 (62)		401 (54)		8.0 [3.0 to 13.0]
Ischemic heart disease	120 (16)		80 (11)		5.2 [1.8 to 8.7]
Arrythmias	122 (16)		105 (14)		2.1 [–1.5 to 5.8]
Anticoagulation	113 (15)		78 (11)		4.6 [1.2 to 8.0]
Congestive heart failure	30 (4.0)		13 (1.8)		2.3 [0.5 to 4.1]
Lung disease	133 (18)		131 (18)		0.1 [–3.8 to 4.0]
Pulmonary embolism	26 (3.5)		7 (0.9)		2.5 [1.1 to 4.2]
Deep vein thrombosis	27 (3.6)		10 (1.4)		2.3 [0.7 to 4.0]
Diabetes	93 (13)		65 (8.8)		3.7 [0.5 to 6.8]
Renal insufficiency	82 (11)		45 (6.1)		4.9 [2.1 to 7.8]
Transient ischemic attack	42 (5.6)		48 (6.5)		–0.9 [–3.3 to 1.6]
CNS bleeding	4 (0.5)		5 (0.7)		–0.1 [–1.1 to 0.8]
Inflammatory arthritis	51 (6.8)		50 (6.8)		0.1 [–2.5 to 2.7]
Depression	78 (11)		73 (9.9)		0.6 [–2.5 to 3.7]
Anxiety	62 (8.3)		43 (5.8)		2.5 [–0.1 to 5.1]
Cancer	94 (13)		78 (11)		2.0 [–1.2 to 5.3]
Type of operation					
Knee replacement	480 (64)		476 (65)		–0.2 [–5.0 to 4.7]
Hip replacement	266 (36)		262 (36)		0.2 [–4.7 to 5.0]
Type of anesthesia					
Spinal	691 (93)		659 (89)		3.3 [0.4 to 6.3]
General	55 (7.4)		79 (11)		–3.3 [–6.3 to –0.4]
ASA classification					
Class 1	41 (5.5)		44 (6.0)		–0.5 [–2.9 to 1.9]
Class 2	469 (63)		528 (72)		–8.7 [–13.4 to –3.9]
Class 3	236 (32)		164 (22)		9.4 [4.9 to 13.9]
Class 4	0 (0)		2 (0.3)		–0.3 [–1.0 to 0.3]
ASA ≥ 3	236 (32)		166 (23)		9.1 [4.6 to 13.6]
Hemoglobin, g/L ^a^					
Preoperative ^a^	140.9 (12.6)		141.3 (13.4)	2 (0.3)	–0.4 [–1.8 to 0.9]
Postoperative ^a^	112.9 (12.7)	4 (0.5)	115.9 (12.8)	34 (4.6)	–3.0 [–4.4 to –1.7]
Postoperative drop ^a^	28.0 (8.4)	4 (0.5)	25.8 (10.9)	34 (4.6)	2.2 [1.2 to 3.2]
Anemia preoperative	44 (5.9)	0 (0)	56 (7.6)	2 (0.3)	–1.7 [–4.3 to 0.9]
Mild	38 (5.1)		47 (6.3)		–1.3 [–3.7 to 1.1]
Moderate	6 (0.8)		8 (1.1)		–0.3 [–1.4 to 0.8]
Severe			1 (0.1)		–0.1 [–0.8 to 0.4]
Creatinine, *µ*mol/L ^a^					
Preoperative	83.9 (47.7)		78.5 (22.6)	2 (0.3)	5.4 [2.5 to 10.7]
Postoperative	85.7 (56.0)	5 (0.7)	81.4 (29.3)	34 (4.6)	4.3 [0.6 to 10.3]
Glucose, mmol/L ^a^					
Preoperative	6.4 (1.5)	1 (0.1)	6.1 (1.5)	13 (1.8)	0.3 [0.1 to 0.4]
Postoperative	6.5 (1.2)	12 (1.6)	6.8 (1.3)	38 (5.1)	–0.3 [–0.4 to –0.2]
HbA1c, mmol/L ^a^					
Preoperative	37.8 (7.3)	7 (0.9)	38.1 (7.6)	17 (2.3)	–0.2 [–1.0 to 0.5]
HbA1c for non-DM patients		7 (1.1)			
42–47 mmol/mol	49 (6.6)		52 (7.0)		–0.3 [–3.3 to 2.6]
> 47 mmol/mol	8 (1.1)		13 (1.8)		–0.7 [–2.2 to 0.7]
Albumin, g/L ^a^	43.4 (2.7)	10 (1.3)	44.2 (2.7)	12 (1.6)	–0.7 [–1.0 to –0.5]
Albumin < 35 g/L	1 (0.1)	10 (1.3)	1 (0.1)	12 (1.6)	0.0 [–0.6 to 0.6]
Lymphocytes < 1.5x10⁹/L	79 (11)	1 (0.1)	129 (18)	2 (0.3)	–6.9 [–10.5 to –3.4]
Vitamin D, nmol/L (SD) ^a,b^	84.7 (34.5)	5 (0.7)	79.6 (33.9)	16 (2.2)	5.1 [1.6 to 8.7]
Vitamin D < 50 nmol/L ^c^	16 (2.1)	20 (2.7)	121 (16)	16 (2.2)	–14.6 [–17.6 to –11.7]
Peri- and postoperative differences					
Blood transfusion	35 (4.7)		19 (2.6)		2.1 [0.2 to 4.1]
Length of stay (days) ^a^	1.9 (3.2)		1.7 (1.7)		0.1 [–0.1 to 0.5]
Postoperative medical complications ≤ 6 weeks from operation					
Myocardial infarction			2 (0.3)		
Heart failure	6 (0.8)		3 (0.4)		0.4 [–0.5 to 1.4]
Pneumonia	3 (0.4)		7 (0.9)		–0.5 [–1.6 to 0.4]
Transient ischemic attack	3 (0.4)		7 (0.9)		–0.5 [–1.6 to 0.4]
Pulmonary embolism	5 (0.7)		3 (0.4)		0.3 [–0.6 to 1.2]
Deep vein thrombosis	3 (0.4)		3 (0.4)		0.0 [–0.8 to 0.8]
Renal insufficiency	24 (3.2)		11 (1.5)		1.7 [0.2 to 3.4]
Death	0 (0.0)		0 (0.0)		0.0 [–0.5 to 0.5]

a Values are mean (SD)

b Vitamin D results for the intervention group are based on blood tests taken on decision for operation.

c Results for number of patients with vitamin D < 50 nmol/L 1 week prior to operation in the intervention group are based on repeated blood tests of those 91 patients who had < 50 nmol/L on decision for operation.

Results from the questionnaire 1 week prior to operation showed that the intervention group had a substantially higher level of awareness of modifiable risk factors than the control group and tried to a higher extent to influence them. A high ratio of patients followed the planned optimization pathway and visited their primary care physician after preoperative evaluation, i.e., 93%, but only 7.9% in the control group (difference 85%, CI 82 to 88). The intervention arm also more often visited other healthcare providers before the operation (Table 3, see Appendix). The intervention group was either satisfied or very satisfied with the hospital service, i.e., 65% (missing answers 13%), while this was 34% for primary care (missing answers 48%). Only 15% in the intervention group considered themselves either aware or well aware of the cooperation between hospital and primary care during the wait for operation, with 30% missing answers (Table 4, see Appendix).

**Table 3:** Answers to questionnaire by control and intervention groups at preoperative visit 1 week prior to operation. Difference between the 2 groups is shown with 95% confidence interval. Values are count (%)

Answers	Intervention group			Yes	Control group Not applicable	Missing	Difference [CI]
	Yes	Not applicable	Missing				
*During wait for surgery, have you been informed of the following health factors and possible influence of them on the outcome of the operation?*							
Weight	613 (82)	35 (4.7)	23 (3.1)	321 (44)	130 (18)	21 (2.8)	38.7 [34.0 to 43.1]
Motion	646 (87)	13 (1.7)	26 (3.5)	381 (52)	112 (15)	18 (2.4)	35.0 [30.5 to 39.2]
Nutrition	591 (79)	19 (2.5)	49 (6.6)	281 (38)	138 (19)	27 (3.7)	41.1 [36.5 to 45.6]
Smoking	355 (48)	276 (37)	48 (6.4)	182 (25)	320 (43)	27 (3.7)	22.9 [18.1 to 27.6]
Anemia	260 (35)	191 (26)	106 (14)	83 (11)	257 (35)	59 (8.0)	23.6 [19.4 to 27.7]
*During wait for surgery, have you tried to influence the following health factors?*							
Weight	524 (70)	32 (4.3)	38 (5.1)	440 (60)	54 (7.3)	31 (4.2)	10.6 [5.8 to 15.4]
Motion	590 (79)	17 (2.3)	31 (4.2)	500 (68)	45 (6.1)	27 (3.7)	11.3 [6.9 to 15.8]
Use of							
primary care physical activity	83 (11)		482 (65)	42 (5.7)		486 (66)	5.4 [2.6 to 8.3]
physiotherapy	270 (36)		347 (47)	201 (27)		388 (53)	9.0 [4.2 to 13.6]
Motion incidences/week			163 (22)			71 (9.6)	
0	66 (8.8)			114 (15)			–6.6 [–9.9 to –3.3]
1	39 (5.2)			49 (6.6)			–1.4 [–3.9 to 1.0]
2	93 (13)			131 (18)			–5.3 [–8.9 to –1.6]
3	159 (21)			158 (21)			–0.1 [–4.3 to 4.1]
4	115 (15)			93 (13)			2.8 [–0.7 to 6.4]
≥ 5	111 (15)			122 (17)			–1.7 [–5.4 to 2.1]
Motion length, minutes			168 (23)			78 (11)	
0	58 (7.8)			101 (14)			–5.9 [–9.1 to –2.8]
0–20	121 (16)			117 (16)			0.4 [–3.4 to 4.1]
20–40	222 (30)			200 (27)			2.7 [–1.9 to 7.2]
40–60	132 (18)			170 (23)			–5.3 [–9.4 to –1.2]
> 60	45 (6.0)			72 (9.8)			–3.7 [–6.5 to –1.0]
Nutrition	529 (71)	23 (3.1)	52 (7.0)	440 (60)	57 (7.7)	34 (4.6)	11.3 [6.5 to 15.8]
Smoking	142 (19)	422 (57)	92 (12)	102 (14)	450 (61)	66 (8.9)	5.2 [1.4 to 9.0]
Still smoking at time of surgery	28 (3.8)		10 (1.3)	54 (7.3)		11 (1.5)	–3.6 [–6.0 to –1.2]
*If previous smoker, when did you quit?*			427 (57)			434 (59)	
< 6 weeks	16 (2.1)				6 (0.8)		1.3 [0.1 to 2.7]
6 week to < 6 moths	13 (1.7)				6 (0.8)		0.9 [–0.3 to 2.2]
6 months to < 1 year	15 (2.0)				6 (0.8)		1.2 [0.0 to 2.6]
≥ 1 year	275 (37)				286 (39)		–1.9 [–6.8 to 3.0]
Anemia treated with iron supplement	61 (8.2)	259 (35)	97 (13)	22 (3.0)	323 (44)	90 (12)	5.2 [2.9 to 7.6]
*During wait for surgery, have you been in contact with other healthcare providers?*							
Primary care	695 (93)			58 (7.9)		102 (14)	85.3 [82.3 to 87.7]
Cardiologist	205 (28)			90 (12)	147 (20)	77 (10)	15.3 [11.3 to 19.2]
Pulmonologist	23 (3.1)	153 (21)	217 (29)	25 (3.4)	180 (24)	90 (12)	–0.3 [–2.2 to1.5]
Endocrinologist	23 (3.1)	167 (22)	222 (30)	14 (1.9)	206 (28)	90 (12)	1.2 [–0.4 to 2.9]
Nephrologist	21 (2.8)	166 (22)	222 (30)	9 (1.2)	207 (28)	93 (13)	1.6 [0.1 to 3.2]

**Table 4:** Answers by individuals in intervention group to questionnaire at preoperative visit. Values are count (%)

Answers	Yes	Missing
Appreciation of hospital service		96 (13)
1. Very dissatisfied	100 (13)	
2. Dissatisfied	13 (1.7)	
3. Neutral	53 (7.1)	
4. Satisfied	219 (29)	
5. Very satisfied	265 (36)	
Appreciation of primary care service		355 (48)
1. Very dissatisfied	30 (4.0)	
2. Dissatisfied	10 (1.3)	
3. Neutral	98 (13)	
4. Satisfied	144 (19)	
5. Very satisfied	109 (15)	
Noticed hospital and primary health		
care cooperation		224 (30)
1. Did not notice	146 (20)	
2. Vaguely noticed	78 (11)	
3. Noticed	183 (25)	
4. Aware	82 (11)	
5. Well aware	33 (4.4)	

### Surgical site complications

In both groups over 95% attended a follow-up visit at the hospital orthopedic outpatient clinic 6 weeks after operation; others were followed by primary care, were admitted to hospital at the time, or received a phone call from the surgeon. No patient was lost to follow-up.

All complications gathered from the surgical site were lower in the intervention group at 11.3% versus 15.7% (difference –4.5%, CI –8.0 to –1.0) (Table 5). Superficial SSIs were less common in the intervention group at 5.6% versus 7.7% (difference –2.1%, CI –4.7 to 0.4). PJI developed in all cases from superficial SSI and occurred in 1.5% of cases in the intervention group but 0.9% in the control group (difference 0.5%, CI –0.7 to 1.8).

**Table 5:** Comparison of surgical site complications between control and intervention groups within 6 weeks after operation. The difference in proportions between the 2 groups is shown with 95% confidence interval. Values are count (%)

Surgical site complications	Intervention	Missing	Control	Missing	Difference % [CI]
Any surgical site complications	84 (11)	0	116 (16)	3 (0.4)	–4.5 [–8.0 to –1.0]
Superficial SSI	42 (5.6)	0	57 (7.7)	3 (0.4)	–2.1 [–4.7 to 0.4]
Drainage	37 (5.0)	0	58 (7.9)	3 (0.4)	–2.9 [–5.5 to –0.4]
Bleeding	36 (4.8)	0	42 (5.7)	3 (0.4)	–0.9 [–3.2 to 1.4]
Dehiscence	11 (1.5)	0	12 (1.6)	3 (0.4)	–0.2 [–1.5 to 1.2]
Limb hematoma	23 (3.1)	0	13 (1.8)	3 (0.4)	1.3 [–0.3 to 3.0]
Periprosthetic joint infection	11 (1.5)	0	7 (0.9)	3 (0.4)	0.5 [–0.7 to 1.8]

Regarding superficial SSI, staphylococci were the pathogen in 86% of cases in the intervention group and 95% in the control group (Table 6, see Appendix).

**Table 6:** Positive bacterial cultures in superficial surgical site infections (SSI) and periprosthetic joint infections (PJI). Values are count (%)

Factor	Intervention group (n = 746)	Control group (n = 738)
**Superficial SSI**	42 (5.6)	57 (7.7)
Cultures taken	20/42 (48)	28/57 (49)
Cultures positive	15/20 (75)	18/28 (64)
Bacteria considered the main cause of infection		
Staphylococci		
* S. aureus*	9	10
* S. epidermidis*	4	3
Coagulase negative s.	1	3
* S. caprae*		1
* Pseudomonas*		1
* Enterococcus faecalis*	1	
Additional bacteria cultured		
* Enterococcus faecalis*		1
Corynebacter		1
* Proteus mirabilis*		1
* Escherisia coli*		1
* Streptococcus agalactiae*		1
Enterobacter	1	
No. of patients with		
multibacterial infection	1	3
**PJI**	11 (1.5)	7 (0.9)
Cultures taken	11/11	7/7
Cultures positive	11/11	7/7
Bacteria considered the main cause of infection		
Staphylococci		
* S. aureus*	6	3
* S. epidermidis*	3	1
* S. caprae*		1
* Streptococcus agalactiae*		1
* Proteus mirabilis*		1
Acinetobacter B.	1	
* Enterococcus fecalis*	1	
Additional bacteria cultured		
* S. lugudensis*		1
* Corynebacterium tuberculosteanicum*	1	
* Dermabacter hominis*		1
* Acinetobacter pitti*		1
* S. epidermidis*	2	1
* Micrococcus luteus*	1	
Corynebacterium	1	
Brevibacterium	1	
* Aerococcus viridans*	1	
Dermabacter	1	
No. of patients with		
multibacterial infection	3	2

A univariable logistic regression analysis in the intervention group was unable to establish an association between superficial SSI and the presence of known preoperative risk factors (anemia, HbA1c > 42 mmol/mol, BMI ≥ 30, smoking, vitamin D < 50 nmol/L, weekly motion occasions < 2) 1 week prior to operation (Table 7).

**Table 7:** Odds ratio (OR) and 95% confidence interval (CI) of superficial surgical site infections (SSI) for patients in the intervention group with preoperative risk factors 1 week prior to operation

Risk factor	OR [CI]	Missing
Preoperative anemia	0.79 [0.18–3.37]	0
HbA1c ≥ 42 mmol/mol	1.13 [0.51–2.51]	7
BMI at operation ≥ 30	1.20 [0.64–2.27]	0
Smoking	2.12 [0.61–7.32]	10
Vitamin D < 50 nmol/L	2.53 [0.55–11.5]	20
Weekly motion ≤ 2	1.52 [0.72–3.20]	163

In a multivariable logistic regression analysis adjusting for ASA classification (reflecting comorbidities), age, and sex, being in the intervention group was associated with lower odds of superficial SSI compared with being in the control group (OR 0.64, CI 0.42–0.97) (Table 8).

**Table 8:** Adjusted and crude odds ratio (OR) and 95% confidence interval (CI) of occurrence of superficial surgical site infections in all patients according to preoperative risk factors 1 week prior to operation. 3 observations were deleted due to being missing. Adjustments were made for ASA class II, age, and sex

Risk factor	Adjusted OR [CI]	Crude OR [CI]
Group: intervention	0.64 [0.42–0.97]	0.71 [0.40–1.07]
ASA class II or above	1.83 [1.17–2.82]	
Age	1.02 [0.99–1.04]	
Male sex	1.67 [1.11–2.54]	

## Discussion

The aim of our study was to explore whether preoperative optimization of modifiable risk factors could mitigate infectious outcomes after TJA. We found that with structured cooperation between hospital and primary health care it is possible to utilize the time while waiting for operation to improve patients’ modifiable risk factors before TJA and reduce the odds of developing superficial SSI compared with a control group receiving a conventional preoperative preparation.

The choice to use superficial SSI as a primary outcome instead of PJI, which we did because of a low number of patients, may be considered controversial. However, studies have shown that patients developing superficial SSI after primary elective hip or knee arthroplasty have a high risk of progressing into PJI as was found in a recent study where 29% of patients with superficial SSI progressed to PJI [16], and others have shown superficial SSI to increase the risk of PJI 35-fold [17]. Risk of development of superficial SSI has been associated with BMI ≥ 30, HbA1c ≥42, and ASA classification ≥ 3 [11,16], which have also been associated with the development of PJI [18]. In our study, PJI occurring within 6 weeks after operation developed in all cases from superficial SSI. Factors influencing superficial SSI to develop into PJI are not only patient dependent but also rely on the virulence of the microorganisms present, which may cause some variation in PJI incidence [19,20].

### Effect of optimization in the intervention group waiting for operation

The high awareness and will to influence modifiable risk factors (Table 3, see Appendix) and the general improvement in them while waiting for operation (Table 1) resulted after a rather straightforward optimization pathway, where 93% of the intervention group visited their primary care physician, and those with BMI ≥ 40 and vitamin D deficiency received advice from a hospital-based clinical nutritionist and also consultations from other specialists when deemed necessary. In a recent literature review of preoperative optimization of modifiable risk factors involving 69 studies, only 3 randomized controlled trials and 8 prospective cohort studies were included. Many of the studies mainly observed only 1 modifiable risk factor [1] making comparison with our study somewhat difficult. However, in general, studies on preoperative optimization have shown improvement in SSI [21], PJI [6], total complication rate [21], length of stay [5,22], postoperative emergency ward visits [22], hospital readmissions [7], costs [23], and patient engagement [23] to a varying extent. This is in line with our study, where we have shown increased patient engagement and beneficial effects on total complication and superficial SSI rates.

### Comparison of intervention and control group

We did not find any significant association between known modifiable risk factors and superficial SSI in the intervention group. However, in the control group, an association was found with elevated BMI ≥ 30 and HbA1c ≥ 42 mmol/mol [11]. It might be interpreted that by preoperative optimization the associations with these factors in the intervention group were prevented, even though they might be considered at higher risk of complications because of the higher ratio of various comorbidities, also followed by significantly lower odds of developing superficial SSI in the intervention group. This might be explained by the fact that a multidisciplinary approach was used to affect modifiable risk factors, which has been pointed out by others to be necessary for preoperative optimization [24]. Regarding PJI we showed no difference, though our study was not powered to analyze this, but ratios in both groups, i.e., superficial SSI and PJI, are comparable to previous studies [16,25,21].

### Limitations

The primary limitation of the study includes a small patient sample in both the intervention and control arms. Additionally, the COVID-19 pandemic affected the execution of the study and slowed down the inclusion of patients, and might have influenced selection of patients into the study, but it is difficult to predict in what manner. The higher ratio of comorbidities in the intervention group may possibly be explained by that the control group was included 1 week prior to operation and no commitment by the patient was required. The intervention group was included 6–12 months prior to operation and were offered more contact with the healthcare system but at the same time this also possibly required some commitment from the patient. Therefore, patients with known underlying diseases may have been more willing to participate in the study. Also, a possible higher awareness and fear among patients and physicians in the intervention group of superficial SSI could have increased the number of diagnoses of such infection.

### Conclusion

We were able to increase patients’engagement and improve their modifiable risk factors with a structured optimization pathway by cooperation between hospital and primary care. The overall surgical site complication rate was decreased and the odds of developing superficial SSI were reduced in the intervention group.

In perspective, optimizing patients before surgery seems logical but interventions should be studied and their efficacy evaluated.

## Notes

MS: study initiation and design, data collection, data analysis, manuscript writing. MIS, RDV: data analysis, interpretation of data, manuscript writing. YO, IG: study design, interpretation of data, manuscript writing. ELS: interpretation of data, manuscript writing. SK: study initiation and design, data analysis and interpretation, manuscript writing.

Handling co-editors: Marianne Westberg and Robin Christensen

*Acta* thanks Finnur Snorrason and Ricardo Sousa for help with peer review of this manuscript.

## References

[1] MacMahon A, Rao S S, Chaudhry Y P, Hasan S A, Epstein J A, Hegde V, et al. Preoperative patient optimization in total joint arthroplasty – the paradigm shift from preoperative clearance: a narrative review. HSS J 2022; 18: 418-27. doi: 10.1177/15563316211030923. 35846267 PMC9247589

[2] Moyer R, Ikert K, Long K, Marsh J. The value of preoperative exercise and education for patients undergoing total hip and knee arthroplasty: a systematic review and meta-analysis. JBJS Rev 2017; 5: e2. doi: 10.2106/JBJS.RVW.17.00015. 29232265

[3] Premkumar A, Kolin D A, Farley K X, Wilson J M, McLawhorn A S, Cross M B, et al. Projected economic burden of periprosthetic joint infection of the hip and knee in the United States. J Arthroplasty 2021; 36: 1484-9.e3. doi: 10.1016/j.arth.2020.12.005. 33422392

[4] Xu Y, Huang T B, Schuetz M A, Choong P F M. Mortality, patient-reported outcome measures, and the health economic burden of prosthetic joint infection. EFORT Open Rev 2023; 8: 690-7. doi: 10.1530/EOR-23-0078. 37655835 PMC10548306

[5] Bernstein D N, Liu T C, Winegar A L, Jackson L W, Darnutzer J L, Wulf K M, et al. Evaluation of a preoperative optimization protocol for primary hip and knee arthroplasty patients. J Arthroplasty 2018; 33: 3642-8. doi: 10.1016/j.arth.2018.08.018. 30201213

[6] Bullock M W, Brown M L, Bracey D N, Langfitt M K, Shields J S, Lang J E. A bundle protocol to reduce the incidence of periprosthetic joint infections after total joint arthroplasty: a single-center experience. J Arthroplasty 2017; 32: 1067-73. doi: 10.1016/j.arth.2016.11.028. 27956126

[7] Olsen A S, Giunta N M, Jamison M P, Chen A F, Fitz W, Iorio R. A total knee arthroplasty preoperative optimization program managed by an advanced practice provider (physician assistant) decreases complications and cost: a pilot study. J Arthroplasty 2023; 38: S77-S80. doi: 10.1016/j.arth.2023.03.064. 37001621

[8] Indelli P F, Iannotti F, Ferretti A, Valtanen R, Prati P, Perez Prieto D, et al. Recommendations for periprosthetic joint infections (PJI) prevention: the European Knee Associates (EKA) – International Committee American Association of Hip and Knee Surgeons (AAHKS) – Arthroplasty Society in Asia (ASIA) survey of members. Knee Surg Sports Traumatol Arthrosc 2022; 30: 3932-43. doi: 10.1007/s00167-021-06742-1. 34518895

[9] Johns W L, Layon D, Golladay G J, Kates S L, Scott M, Patel N K. Preoperative risk factor screening protocols in total joint arthroplasty: a systematic review. J Arthroplasty 2020; 35: 3353-63. doi: 10.1016/j.arth.2020.05.074. 32600816

[10] McLaughlin J, Scott L J, Owens L, McLeod H, Sillero-Rejon C, Reynolds R, et al. Evaluating a pre-surgical health optimisation programme: a feasibility study. Perioper Med (Lond) 2022; 11: 21. doi: 10.1186/s13741-022-00255-2. 35733182 PMC9219203

[11] Sigurdardottir M, Sigurdsson M I, Olafsson Y, Sverrisdottir S H, Gunnarsdottir I, Sigurdsson E L, et al. Prevalence of modifiable risk factors in primary elective arthroplasty and their association with infections. Acta Orthop 2023; 94: 38-44. doi: 10.2340/17453674.2023.8480. 36727913 PMC9893833

[12] van Groningen L, Opdenoordt S, van Sorge A, Telting D, Giesen A, de Boer H. Cholecalciferol loading dose guideline for vitamin D-deficient adults. Eur J Endocrinol 2010; 162: 805-811. doi: 10.1530/EJE-09-0932. 20139241

[13] Thorsdottir I, Eriksen B, Eysteinsdottir S. Nutritional status at submission for dietetic services and screening for malnutrition at admission to hospital. Clin Nutr 1999; 18: 15-21. doi: 10.1016/s0261-5614(99)80044-2. 10459078

[14] Surgical site infection event (SSI). National Healthcare Safety Network. Centers for Disease Control and Prevention, 2022. Available from: https://wwwcdcgov/nhsn/pdfs/pscmanual/9pscssicurrentpdf (accessed June 19, 2024)

[15] Parvizi J, Tan T L, Goswami K, Higuera C, Della Valle C, Chen A F, et al. The 2018 definition of periprosthetic hip and knee infection: an evidence-based and validated criteria. J Arthroplasty 2018; 33: 1309-14.e12. doi: 10.1016/j.arth.2018.02.078. 29551303

[16] Eriksson H K, Lazarinis S. Patient-related factors associated with superficial surgical site infection and progression to a periprosthetic joint infection after elective primary total joint arthroplasty: a single-centre, retrospective study in Sweden. BMJ Open 2022; 12: e060754. doi: 10.1136/bmjopen-2022-060754. PMC9486361 36123083

[17] Saleh K, Olson M, Resig S, Bershadsky B, Kuskowski M, Gioe T, et al. Predictors of wound infection in hip and knee joint replacement: results from a 20 year surveillance program. J Orthop Res 2002; 20: 506-515. doi: 10.1016/S0736-0266(01)00153-X. 12038624

[18] Zhong J, Wang B, Chen Y, Li H, Lin N, Xu X, et al. Relationship between body mass index and the risk of periprosthetic joint infection after primary total hip arthroplasty and total knee arthroplasty. Ann Transl Med 2020; 8: 464. doi: 10.21037/atm.2020.03.112. 32395508 PMC7210163

[19] Aggarwal V K, Rasouli M R, Parvizi J. Periprosthetic joint infection: current concept. Indian J Orthop 2013; 47: 10-17. doi: 10.4103/0019-5413.106884. 23531512 PMC3601222

[20] Kim K, Zhu M, Coleman B, Munro J T, Young S W. Differing microorganism profile in early and late prosthetic joint infections following primary total knee arthroplasty - implications for empiric antibiotic treatment. J Arthroplasty 2022; 37: 1858-64 e1851. doi: 10.1016/j.arth.2022.04.014. 35460813

[21] Nussenbaum F D, Rodriguez-Quintana D, Fish S M, Green D M, Cahill C W. Implementation of preoperative screening criteria lowers infection and complication rates following elective total hip arthroplasty and total knee arthroplasty in a veteran population. J Arthroplasty 2018; 33: 10-13. doi: 10.1016/j.arth.2017.07.031. 28838614

[22] Dlott C C, Moore A, Nelson C, Stone D, Xu Y, Morris J C, et al. Preoperative risk factor optimization lowers hospital length of stay and postoperative emergency department visits in primary total hip and knee arthroplasty patients. J Arthroplasty 2020; 35: 1508-15.e2. doi: 10.1016/j.arth.2020.01.083. 32113812

[23] Kim K Y, Anoushiravani A A, Chen K K, Li R, Bosco J A, Slover J D, et al. Perioperative orthopedic surgical home: optimizing total joint arthroplasty candidates and preventing readmission. J Arthroplasty 2019; 34: S91-S96. doi: 10.1016/j.arth.2019.01.020. 30745217

[24] Dietz M J, Chaharbakhshi E O, Roberts A J, Gilligan P H, Kasicky K R, Pincavitch J D. Maintenance of surgical optimization in total joint arthroplasty patients. J Arthroplasty 2024; 39: 1650-55.e1. doi: 10.1016/j.arth.2024.01.013. 38216000

[25] Kong L, Cao J, Zhang Y, Ding W, Shen Y. Risk factors for periprosthetic joint infection following primary total hip or knee arthroplasty: a meta-analysis. Int Wound J 2017; 14: 529-6. doi: 10.1111/iwj.12640. 27397553 PMC7949746

[26] Kunutsor S K, Beswick A D, Whitehouse M R, Blom A W, Lenguerrand E. Implant fixation and risk of prosthetic joint infection following primary total hip replacement: meta-analysis of observational cohort and randomised intervention studies. J Clin Med 2019; 8. doi: 10.3390/jcm8050722. 31117318 PMC6571822

